# Are There Associations between Seminal Plasma Advanced Oxidation Protein Products and Selected Redox-Associated Biochemical Parameters in Infertile Male Patients? A Preliminary Report

**DOI:** 10.3390/cells11223667

**Published:** 2022-11-18

**Authors:** Ewa Janiszewska, Izabela Kokot, Agnieszka Kmieciak, Iwona Gilowska, Ricardo Faundez, Ewa Maria Kratz

**Affiliations:** 1 Department of Laboratory Diagnostics, Division of Laboratory Diagnostics, Faculty of Pharmacy, Wroclaw Medical University, Borowska Street 211A, 50-556 Wroclaw, Poland; 2 Institute of Health Sciences, Collegium Salutis Humanae, University of Opole, Katowicka Street 68, 45-060 Opole, Poland; 3 Clinical Center of Gynecology, Obstetrics and Neonatology in Opole, Reference Center for the Diagnosis and Treatment of Infertility, Reymonta Street 8, 45-066 Opole, Poland; 4 InviMed Fertility Clinics, Rakowiecka Street 36, 02-532 Warsaw, Poland

**Keywords:** male infertility, seminal plasma, oxidative stress parameters, oxidative-antioxidant balance, seminal plasma biochemical parameters

## Abstract

Oxidative stress (OS) is one of the reasons for male infertility. Seminal plasma contains a multitude of enzymes and ions which influence OS and thus may affect male fertility. The aim of the study was to check for associations between seminal plasma advanced oxidation protein products (AOPP) concentrations and levels of selected biochemical parameters (total protein, iron, uric acid, magnesium, calcium) in infertile men, and establish whether they are associated with sperm disorders. Seminal plasma AOPP, as well as total protein, iron, uric acid, calcium, and magnesium concentrations, were determined for the following patient groups: normozoospermic (N; *n* = 33), teratozoospermic (T; *n* = 30), asthenoteratozoospermic (AT; *n* = 18), and oligoasthenoteratozoospermic (OAT; *n* = 28). AOPP concentrations were significantly higher in N and T groups in comparison to AT and OAT groups. Total protein concentrations were significantly lower in the T group in comparison to the AT and OAT groups, whereas iron concentrations significantly decreased in the OAT group in comparison to the T and N patients. AOPP differentiates AT patients from men with other sperm disorders. Our results suggest that asthenozoospermia may be connected with total protein levels. Insufficient iron levels may reflect a decrease in sperm count.

## 1. Introduction

Male infertility alone constitutes approximately 40% of infertility cases [1] and has become a growing problem worldwide [2]. The World Health Organization (WHO) recommends semen analysis, which applies to semen and spermatozoa properties [3], but seminal plasma contains a multitude of proteins, glycoproteins, lipids, enzymes, ions, and other chemical compounds that may influence the proper spermatozoa maturation process, as well as gamete fusion [4,5]. Among possible reasons for male infertility, oxidative stress (OS) is one of the main causes of idiopathic male infertility [6,7]. It has been established that a lack of oxidative-antioxidant balance affects the quality of sperm parameters, such as morphology, motility and viability [8,9].

Oxidative stress is defined as an imbalance between the generation of reactive oxygen species (ROS) and the protective action of antioxidant systems responsible for their neutralization and removal [10]. Imbalance between the production and utilization of ROS leads to the damage of many cell structures, especially the phospholipids of cellular membranes. In turn, lipid peroxidation triggers signaling cascades of the inflammatory processes which promote the peroxidation of lipids, resulting in intracellular oxidative burden. The sequence of events involves lipid peroxidation, loss of membrane integrity with increased permeability, reduced sperm motility, structural DNA damage, and, finally, apoptosis [11]. Sperm cells are mitochondria-rich structures, and these cell organs enable proper sperm motility. In the case of oxidative stress, as a product of nicotinamide adenine dinucleotide (NAD)-dependent redox reactions, ROS damage mitochondria, leading to sperm motility disorders [12,13,14]. The effect of oxidative-antioxidative imbalance is also reflected in the sperm cells’ DNA damage, which affects both single- and double-stranded DNA molecules. These damages have been proven to lead to lowered total sperm count [7,15,16]. Although the antioxidant defense system is active in semen, its activity is limited, as the amount of cytoplasm in the sperm cell is low [17]. Spermatozoa are extremely vulnerable to oxidative stress because they lack the necessary repair systems and are unable to restore oxidative damages. Spermatozoa membranes are rich in polyunsaturated fatty acids, which makes them highly susceptible to lipid peroxidation. Oxidative stress results in axonemal damage, decreased sperm viability, and increased midpiece sperm morphological defects. These dysfunctions may contribute to decreased sperm motility [18].

Human seminal plasma constitutes an antioxidant system including both enzymatic and non-enzymatic components. Superoxide dismutase (SOD), catalase (CAT), and glutathione peroxidase (GPX) belong to the main enzymatic antioxidant defense. Low molecular weight non-enzymatic antioxidants such as vitamins (A, E, C, B complex), ions (calcium, iron, zinc, selenium, copper, chrome), glutathione, pantothenic acid, carnitine, and coenzyme Q10 support the enzymatic group of an antioxidant system [19,20,21].

Seminal plasma contains a multitude of protein compounds that may take part in the inactivation of reactive species generated during oxidative stress; as a result, they are oxidized themselves. One of the parameters enabling the measurements of such compounds are advanced oxidation protein products (AOPP) [22]. Other important factors associated with the proper oxidative-antioxidant balance are, i.a., iron (Fe), uric acid (UA), magnesium (Mg), and calcium (Ca), which have been chosen for this study. It has been described that, in the case of cellular metabolism altered by oxidative stress, iron takes part in the lipid membranes’ peroxidation [23]. It is also considered a potent pro-oxidant when not stored in transferrin or ferritin [24]. Uric acid is a product of purine metabolism and belongs to the main low molecular weight antioxidants. UA is a scavenger of peroxyl radicals, hydroxyl radicals, and singlet oxygen generated during OS [25]. Magnesium is the fourth most abundant cation in the human body, and acts as a cofactor of 300 enzymes, i.a., those engaged in protein synthesis, glycolysis, and the transmembrane transport of ions [26]. It is also responsible for reactions with ATP, as well as competing with calcium for binding sites on proteins and membranes [27]. It has been reported that magnesium may be a marker of the prostate gland, and reduced seminal magnesium levels may be associated with premature ejaculation [28]. It has also been reported that hypomagnesemia is associated with elevated lipoprotein oxidation and, via calcium overload, also contributes to an increase in protein oxidation [29]. Calcium plays a crucial role in the acrosome reaction during the fertilization process [4], as well as in determining proper sperm motility [28].

The literature data concerning the basic biochemical laboratory parameters in the oxidative stress context performed in human seminal plasma are insufficient. Therefore, we decided to assess seminal plasma advanced oxidation protein products (AOPP) as well as levels of selected biochemical parameters (total protein (TP), iron (Fe), uric acid (UA), magnesium (Mg), and calcium (Ca)) which may have an impact on the oxidative-antioxidant balance. We were interested in whether they correlated with each other and additionally whether they were associated with disorders of sperm parameters.

## 2. Materials and Methods

### 2.1. Patient Samples

Seminal plasma samples were collected from infertile male patients who attended the Clinical Center of Gynecology, Obstetrics and Neonatology in Opole (Poland) and Fertility Clinics InviMed in Warsaw (Poland). The informed consent was signed by all patients that participated in this study. Our study was conducted according to the guidelines of the Helsinki II declaration, and the protocol was approved by the Bioethics Human Research Committee of Wroclaw Medical University (No. KB 549/2019 and No. KB 707/2019).

Seminal samples were collected into sterile containers after 3–5 days of sexual abstinence (inclusion criterium) through masturbation. After liquefaction (maximum 60 min at 37 °C), standard semen analysis was performed according to WHO 2010 directives (WHO 2010). Semen volume, pH, and sperm viability were assessed using manual techniques, whereas total sperm count in the ejaculate, sperm concentration, total motility, progressive motility, and morphology were carried out using computer-assisted sperm analysis (SCA Motility and Concentration, software version 6.5.0.5, Microptic SL, Barcelona, Spain). All input data in this method were consistent with current WHO recommendations for semen analysis. Next, the ejaculates were centrifuged at 3500× *g* for 10 min at room temperature, and the supernatants were aliquoted and stored at −86 °C in the Wroclaw Medical University Biobank until use.

Based on standard semen analysis (sperm concentration, progressive motility and morphology of spermatozoa), seminal plasma samples (*n* = 109) were divided into groups: normozoospermic (N, *n* = 33; normal values of ejaculate parameters; median age: 32 years [IQR 24–49]), teratozoospermic (T, *n* = 30; <4% of spermatozoa had normal morphology; median age: 33 years [IQR 28–36]), asthenoteratozoospermic (AT, *n* = 18; <32% of sperm demonstrated progressive motility and <4% of spermatozoa had normal morphology; median age: 34 years [IQR 31–36]) and oligoasthenoteratozoospermic (OAT, *n* = 28; sperm count <15 × 10^6^ mL^–1^, <32% of sperm demonstrated progressive motility and <4% of spermatozoa had normal morphology; median age: 32 years [IQR 30–35]). None of the seminal samples were infected by bacteria and/or leukospermic. Active inflammation manifested by elevated serum C-reactive protein levels was also an exclusion criterion.

### 2.2. AOPP Determination

AOPP determination, based on the redox reaction, was carried out according to Witko-Sarsat et al. [30], with the modifications described below. Advanced Oxidation Protein Products present in seminal plasma react with potassium iodide solution (KI) in the presence of acetic acid solution, producing the reduced form of oxidation protein products and iodate ions [21,31]. Briefly, 10 µL of KI was added directly into the well of ELISA plate to 200 µL of twenty-fold diluted in phosphate-buffered saline (PBS) seminal plasma sample, incubated for 2 min at room temperature, and mixed with 20 µL of glacial acetic acid. Absorbance was measured immediately at 340 nm against a blank sample without biological material but containing all other reagents, using the Multiskan Go ELISA plate reader (Thermo Fischer Scientific, Roskilde, Denmark). The obtained results were expressed in chloramine T concentrations which served as a standard, containing parallel chemical moiety as proteins present in seminal plasma. A calibration curve was constructed for chloramine T concentrations ranging from 0 to 80 µmol/L. Blood serum samples with known AOPP concentrations were used as measurement controls for each experiment. All determinations were performed in duplicate to minimize measurement imprecision, using ELISA plates (Nunc MaxiSorp, Thermo Fisher Scientific, Roskilde, Denmark) to reduce the volume of samples used for analysis.

### 2.3. Biochemical Parameters Measurement

The concentrations of total protein, iron, uric acid, calcium, and magnesium were measured using the biochemical autoanalyzer Konelab20i^®^ (Thermo Scientific, Vantaa, Finland). To determine the seminal plasma total protein concentrations, the biuret method was used (Total Protein Plus, Thermo Scientific, catalog No. 981826, Vantaa, Finland) according to the manufacturer’s instruction. Briefly, protein and copper ions in alkaline solutions formed a colored complex, and the absorbance of the formed complex was subsequently measured at 540 nm. The method used EDTA as a chelating and stabilizing agent for copper ions. Iron concentrations were measured using the colorimetric method with Ferene-S (Iron, Thermo Scientific, catalog No. 981236, Vantaa, Finland), following the manufacturer’s instruction. Briefly, in the first step, the iron bonded with proteins was released by guanidine buffer. Then, the total iron (free iron ions as well as iron released from the proteins) reacted with Ferene-S, forming a complex chemical compound, the absorbance of which was read at 600 nm. To measure the uric acid concentrations, a commercial Uric Acid AOX (catalog No. 981391, Thermo Scientific, Vantaa, Finland) reagent was used following the manufacturer’s instruction. The methodology of UA determination used in our study was based on UA oxidation by uricase to allantoin. The generated hydrogen peroxide reacted with 4-aminoantipyrine (4-AAP) and N-ethyl-N-(hydroxy-3-sulfopropyl)-m-toluidine (TOOS), forming a blue–violet product. The absorbance of the formed colored reaction product was measured at 540 nm. Magnesium concentration was measured using xylidyl blue (Magnesium XL FS, DiaSys, catalog No. 146109910021, Holzheim, Germany) according to the manufacturer’s protocol. The absorbance of the purple complex, formed as a product of the reaction, was measured at 540 nm. To determine the calcium concentration, a colorimetric method using arsenazo III was used (Calcium AS FS, DiaSys, catalog No. 11309910021, Holzheim, Germany), following the manufacturer’s instruction. The intensity of the reaction product, the blue complex, was measured at 600 nm.

### 2.4. Statistical Analysis

All statistical calculations were performed using Statistica 13.3 PL software (StatSoft Inc., Tulsa, OK, USA). The normality of distribution for values of all parameters investigated was analyzed with the Shapiro–Wilk test. The obtained values of AOPP and other biochemical parameters’ concentrations were presented as mean ± SD (SD—standard deviation), as well as on the graphs as median with interquartile range (Q1–Q3). As the values of examined parameters did not reach normal distribution, the nonparametric Mann–Whitney U-test was used to compare the levels of determined parameters between examined groups. The associations between values of all parameters were checked by Spearman’s rank correlation. The diagnostic significance of the tested parameters was analyzed using receiver operating characteristic (ROC) curves. The *p*-Values < 0.05 were considered significant.

## 3. Results

**Figure 1:**
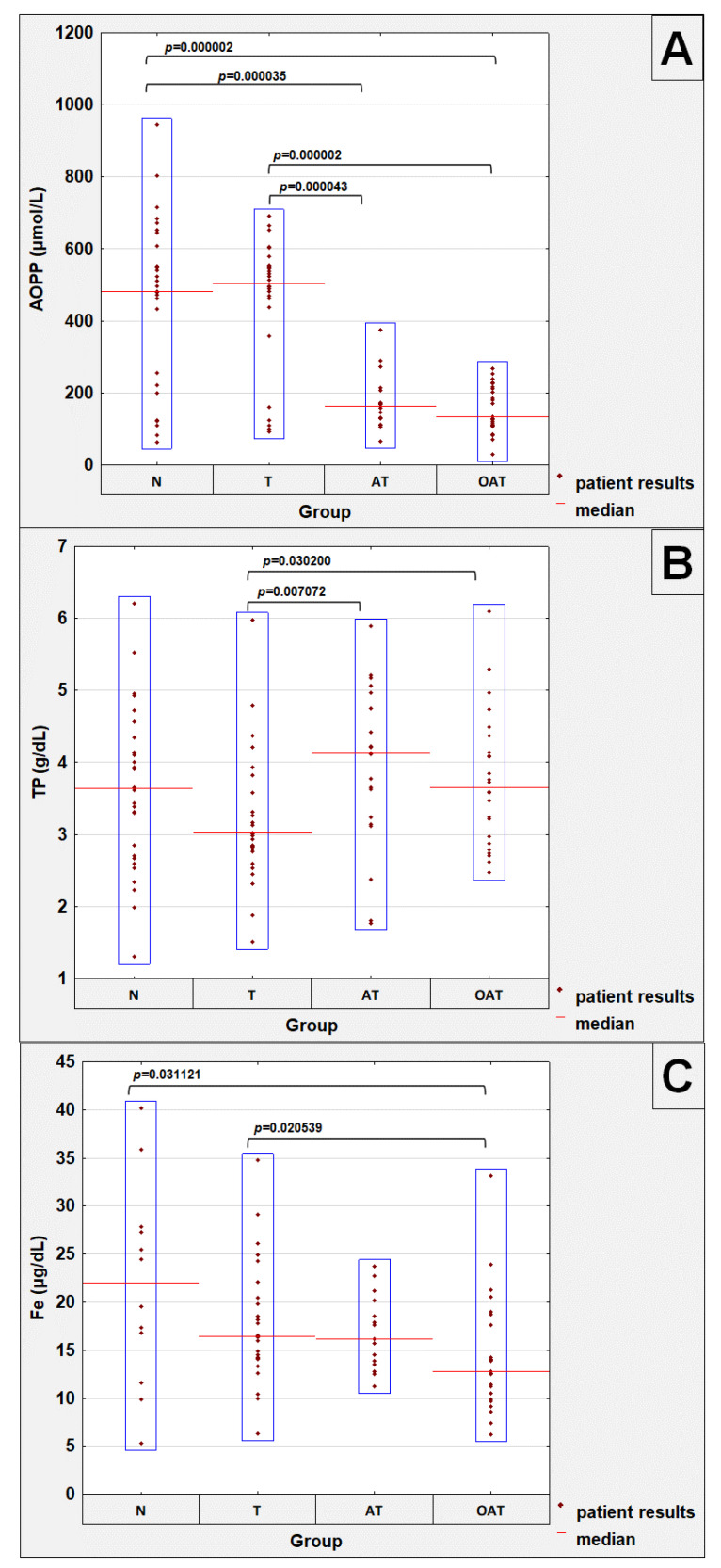
Concentration values of selected seminal plasma parameters which significantly differentiated examined groups of patients: AOPP—advanced oxidation protein products (**A**), TP—total protein (**B**), Fe—iron (**C**). N—normozoospermia, T—teratozoospermia, AT—asthenoteratozoospermia, OAT—oligoasthenoteratozoospermia. All patient results in a given group are gathered in the blue boxes. A two-tailed *p*-Value < 0.05 was considered significant.

The levels of parameters analyzed are presented in Table 1. Significant differences between studied groups are shown in Figure 1.

### 3.1. Seminal Plasma AOPP Levels

Seminal plasma AOPP concentrations were significantly higher in normozoospermic patients (median value: 483.19 µmol/L) when compared to the AT group (median value: 163.51 µmol/L) and OAT (median value: 134.26 µmol/L) groups, with significances of *p* = 0.000035 and *p* = 0.000002, respectively. Moreover, in teratozoospermic patients, seminal plasma AOPP concentrations were also significantly higher (median value: 505.00 µM) in comparison to the AT group (median value: 163.51 µmol/L) and OAT men (median value: 134.26 µmol/L), with significances of *p* = 0.000043 and *p* = 0.000002, respectively (Table 1, Figure 1A).

### 3.2. Seminal Plasma Biochemical Parameters Concentrations

Seminal plasma total protein concentrations were significantly lower in the teratozoospermic group (median value: 3.02 g/dL) in comparison to the AT (median value: 4.13 g/dL) and OAT (median value: 3.66 g/dL) groups, with significances of *p* = 0.007072 and *p* = 0.030200, respectively. No significant differences between the normozoospermic group (median value: 3.64 g/dL) and the other analyzed groups were found (Table 1, Figure 1B).

Seminal plasma iron concentrations were significantly lower in the OAT group (median value: 12.80 µg/dL) when compared to the teratozoospermic (median value: 16.44 µg/dL) and normozoospermic men (median value: 22.01 µg/dL), with significance of *p* = 0.020539 and *p* = 0.031121, respectively. No significant differences between the AT group (median value: 16.15 µg/dL) and the other analyzed groups were found (Table 1, Figure 1C).

No significant differences were found between the groups of men examined for seminal plasma uric acid, magnesium, and calcium concentrations (Table 1). The median value of seminal plasma uric acid concentration was 6.58 mg/dL in normozoospermic patients, 5.84 mg/dL in both teratozoospermic and asthenoteratozoospermic patients, and 6.47 mg/dL in oligoasthenoteratozoospermic patients (Table 1). Median seminal plasma magnesium concentrations in N, T, AT and OAT groups were as follows: 8.25 mg/dL, 7.28 mg/dL, 6.87 mg/dL and 5.80 mg/dL (Table 1). The median value of seminal plasma calcium concentrations was 26.79 mg/dL in the normozoospermic group, 23.58 mg/dL in teratozoospermic men, 18.37 mg/dL in asthenoteratozoospermic patients, and 13.95 mg/dL in the oligoasthenoteratozoospermic group (Table 1).

**Figure 2:**
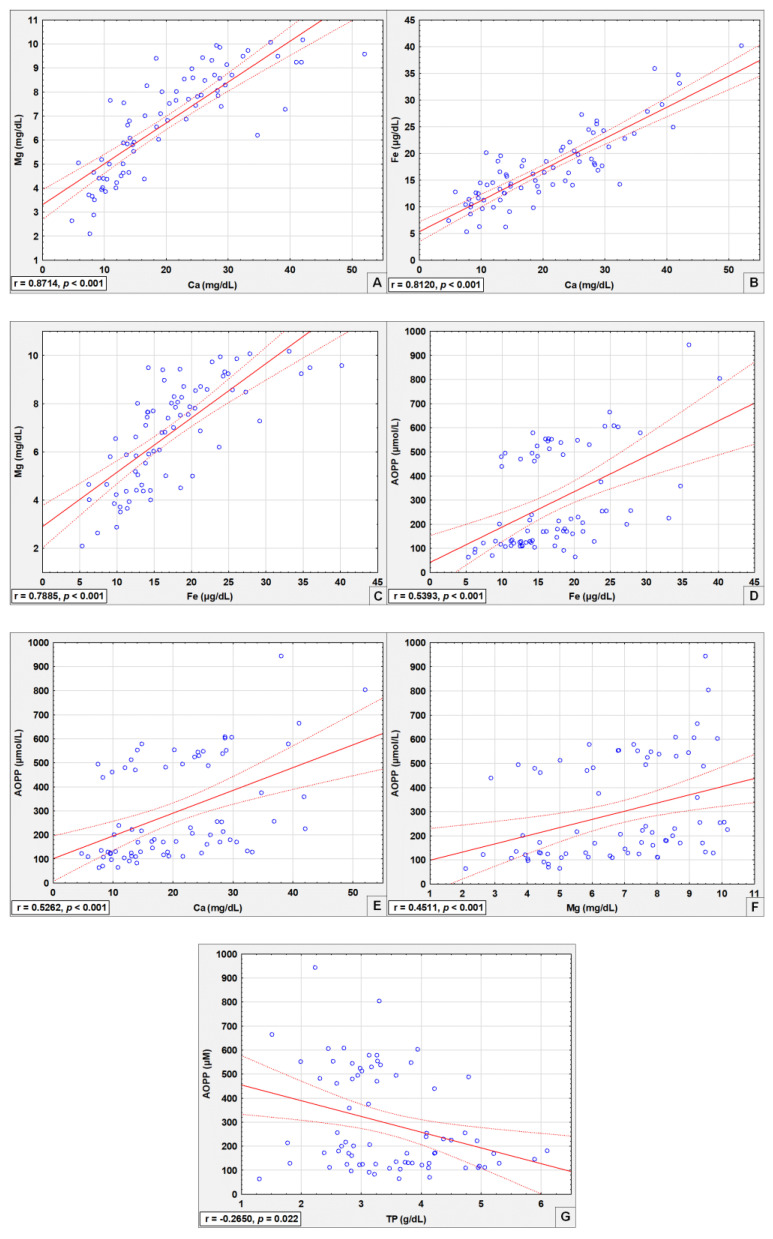
Correlations between concentrations of determined seminal plasma parameters (**A**–**G**). Mg—magnesium, Ca—calcium, Fe—iron, AOPP—advanced oxidation protein products, TP—total protein. The dashed line points indicate the 95% confidence interval. A two-tailed *p*-Value < 0.05 was considered significant.

Significant correlations between the analyzed parameters are shown in Table 2 and Figure 2.

Very strong positive correlations were observed between seminal plasma calcium and magnesium concentrations (*R* = 0.8714, *p* < 0.001; Table 2, Figure 2A), as well as between calcium and iron levels (*R* = 0.8120, *p* < 0.001; Table 2, Figure 2B). Strong positive correlations between seminal plasma magnesium and iron concentrations (*R* = 0.7885, *p* < 0.001; Table 2, Figure 2C) were found. Moderate correlations between AOPP levels and iron (*R* = 0.5393, *p* < 0.001; Table 2, Figure 2D), calcium (*R* = 0.5262, *p* < 0.001; Table 2, Figure 2E), and magnesium (*R* = 0.4511, *p* < 0.001; Table 2, Figure 2F) concentrations were found. Moreover, there were weak negative correlations between AOPP and total protein levels (*R* = −0.2650, *p* = 0.022; Table 2, Figure 2G).

### 3.3. ROC Curves Analysis

**Figure 3:**
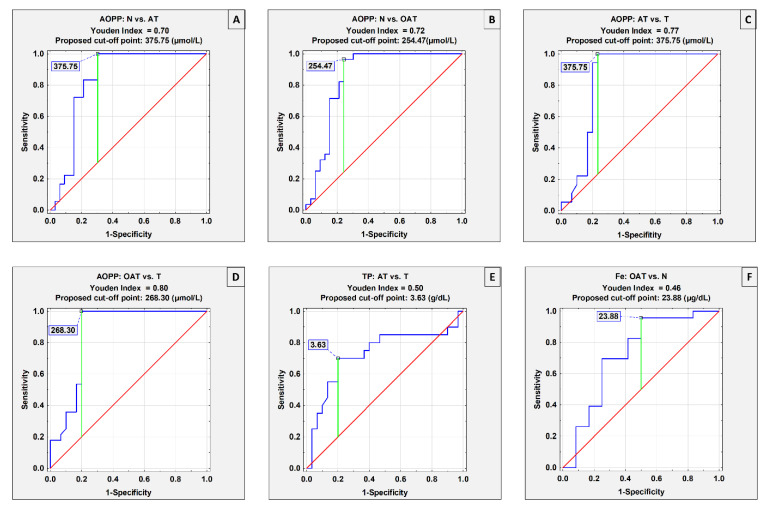
Receiver operating characteristic (ROC) curves for seminal plasma parameters with the area under the curve (AUC) equal to or higher than 0.725 (**A**–**F**). AOPP—advanced oxidation protein products, TP—total protein, Fe—iron. N—normozoospermia, T—teratozoospermia, AT—asthenoteratozoospermia, OAT—oligoasthenoteratozoospermia.

The receiver operating characteristic (ROC) curves analysis was performed only for parameters the levels of which showed significant differences between examined groups (Table 3). Figure 3 presents the results of the ROC curves analysis for parameters for which the area under the curve (AUC) was higher than 0.7. Based on the AUC, the clinical value of laboratory tests can be defined as: 0–0.5–zero, 0.5–0.7–limited, 0.7–0.9–moderate, and >0.9–high [32].

## 4. Discussion

Although seminal plasma AOPP have been investigated before [21,31,33], there is not much information about the associations between the levels of advanced oxidation protein products and concentrations of biochemical parameters in seminal plasma that may have an impact on the oxidative-antioxidant balance in this biological fluid. Physiologically, AOPP are formed throughout an individual’s lifetime in small quantities and increase with age. Significantly higher serum concentrations of AOPP are observed in many pathological conditions. AOPP formation is induced by intensified glycooxidation processes, oxidative-antioxidant imbalance, and coexisting inflammation [34]. The results of our current research may suggest that sperm motility disorders are associated with decreased AOPP levels, which probably interfere with the oxidative-antioxidant balance of seminal plasma. We observed an analogous relationship for the value of the calculated AOPP/TP index (µmol of AOPP per gram of total protein), which was also significantly lower in the seminal plasma of patients with disturbed sperm motility (data not shown). Our previous study revealed that seminal plasma AOPP concentrations were significantly higher in the infertile teratozoospermic and azoospermic patients in comparison to the healthy fertile men [21]. In our other research, seminal plasma AOPP levels were significantly higher in infertile normozoospermic, oligozoospermic, and asthenozoospermic groups than in fertile normozoospermic men [31]. Interestingly, seminal plasma AOPP concentrations investigated by Demir and Ozdem [33] were significantly higher in oligoasthenoteratozoospermic, teratozoospermic, and azoospermic men in comparison to infertile normozoospermic patients. Based on the above information, we presume that the possible association between the formation of advanced oxidation protein products and the disorders of routinely examined sperm parameters is multidirectional and may involve additional factors which were not examined in our study. However, this hypothesis requires verification in future research involving a wider panel of biochemical parameters.

The seminal plasma total protein concentrations found suggest that sperm morphology abnormalities are the only sperm disorder that may be associated with decreased levels of this parameter. In contrast, Collodel et al. [35] reported that decreased seminal plasma total protein concentrations were associated with sperm motility ≤5th centile [35]. Mendeluk et al. [36] investigated whether seminal plasma total protein concentration is involved in the hyperviscosity of human seminal plasma. The authors did not observe significant differences between the analyzed groups (samples were analyzed according to the WHO criteria from 1998 and regarded as hyperviscous when the length of the thread formed on withdrawal of a glass rod exceeded 2 cm) in seminal plasma total protein concentrations [36]. Taking all this information into account, we may conclude that the measurements of seminal plasma total protein concentration are not sufficient for the identification of the cause of male infertility connected with sperm parameter disorders.

We observed the associations between decreased iron concentrations in seminal plasma with simultaneously existing sperm abnormalities, such as lower total sperm count, decreased motility, and morphology disorders. Our findings are quite similar to those obtained by Skandhan et al. [37], who reported a significant decrease in seminal plasma iron levels in asthenozoospermic and azoospermic patients in comparison to normozoospermic fertile men [37], and suggested that sperm motility disorders may be connected with the decrease in seminal plasma iron concentration. Additionally, a study by Jia et al. [38] reported a positive correlation between Fe levels and sperm concentration and total sperm count, and Shukla et al. [39] showed significantly decreased seminal plasma Fe levels in all infertile groups of patients in comparison to healthy men [39]. On the other hand, Pant and Srivastava [40] did not find significant differences in seminal plasma Fe concentrations between the studied azoospermic, oligoasthenozoospermic, oligozoospermic and asthenozoospermic groups [40]. Conversely, seminal plasma iron levels investigated by Marzec-Wróblewska et al. [41] were significantly higher in the teratozoospermic patients in comparison to patients with normal sperm parameters. Inconsistency in the results obtained by the abovementioned authors may be due to differences in the selection of the types of male fertility disorders compared, as well as the methods of iron concentration assessment—many studies concerning iron concentration are based on mass spectrometry (MS) with modifications, whereas our research used spectrophotometry. Magali et al. [42] investigated the role of seminal plasma iron metabolism in oxidative stress, measuring, i.a., seminal plasma iron concentration, but the authors did not refer to the results concerning total seminal plasma iron concentration, suggesting the lack of a relationship between this parameter and other oxidative stress parameters measured [42]. However, the potential pro- and/or antioxidant role of iron in the male reproductive tract is still unknown.

In our study, the mean concentrations of uric acid were higher in the normozoospermic group than in the teratozoospermic group and asthenoteratozoospermic groups, but similar to the oligoasthenoteratozoospermic group; however, the differences were insignificant (Table 1). Lazzarino et al. [43] found no significant differences between groups of infertile patients, also in comparison to the fertile control group. Kanďár et al. [44] investigated seminal plasma uric acid concentrations in smoking and non-smoking infertile men and also found no significant differences in seminal plasma UA levels between the studied groups; however, there is no data concerning the results of routine semen examination of those patients [44]. On the other hand, Allahkarami et al. [45] showed the presence of a negative correlation between seminal plasma uric acid concentration and sperm morphology [45]. Further experiments concerning the role of seminal plasma uric acid may demonstrate the particular role of uric acid in the context of male fertility.

We did not find significant differences in magnesium and calcium concentrations between examined groups of infertile patients; however, the levels of both elements were visibly higher in normozoospermic men than in patients with sperm disorders. Colagar et al. [46] found no significant differences in seminal plasma magnesium and calcium levels between infertile and fertile smoking and non-smoking groups. While Wong et al. [47] found no significant differences between seminal plasma magnesium and calcium concentrations between fertile and infertile men (semen analysis was performed according to WHO guidelines from 1992), the study revealed strong positive correlations between the seminal plasma concentrations of these elements [47], as does our present research. Sørensen et al. [48] investigated whether seminal plasma magnesium and calcium levels, among others, have an impact on time to pregnancy in healthy couples. No significant differences in the levels of these elements were shown for either group: men from couples with short time to pregnancy (one month), and men from couples with long time to pregnancy (ten months). The findings of Abdul-Rasheed [27] revealed significant decreases in seminal plasma magnesium concentrations in all studied infertile groups in comparison to normozoospermic fertile men. Based on the presented information, we may conclude that the role of calcium in the male reproductive tract in the context of maintenance of oxidative-antioxidant balance is complex. Achcińska and Kratz [4] underlined that calcium, especially its sufficient intracellular levels, is crucial in proper capacitation processes, as well as gamete fusion [4]. This raises the question of whether there are associations between seminal plasma and intracellular calcium levels in the groups of infertile men in comparison to healthy controls. Taking this information into account, together with the moderate positive correlation between seminal plasma calcium concentrations and AOPP levels, further investigation is needed in this field, especially with regards the comparison of results obtained for infertile and fertile men.

Our study revealed a very strong positive correlation between seminal plasma calcium and magnesium concentrations (Table 2, Figure 2A), which is consistent with the results of Wong et al. [47] but may be surprising, given the fact that magnesium is a calcium antagonist [49]. It is worth underlining that calcium plays a crucial role in the initiation of acrosomal reaction; moreover, during the capacitation process, sperm cells are modified, and the level of calcium ions physiologically increases [50]. Based on this information, we may presume that abnormal seminal plasma magnesium levels in infertile men may interrupt the proper biological function of calcium. A very strong positive correlation between seminal plasma calcium and iron concentrations was also found (Table 2, Figure 2B). Mitochondrial iron metabolism has been proven crucial for energy production and metabolism during spermatogenesis [51], but the relationship between calcium and iron levels in spermatogenesis should be investigated. Results of our research concerning the relationship between seminal plasma magnesium and iron concentrations contrast with the study results obtained by Srivastava et al. [40], who found a lack of significant correlations between these parameters. Our results, which reveal a strong positive correlation between Mg and Fe levels (Table 2, Figure 2C), seem surprising, given that lipid peroxidation is increased in the case of elevated iron levels and decreased magnesium concentrations [23,29]. Perhaps there is another mechanism that takes part in the regulation of magnesium and iron expression in the male reproductive tract. Moderate positive correlations between seminal plasma AOPP and iron, calcium, and magnesium were found (Table 2, Figure 2D–F), which may confirm the important role of these elements in protein oxidation. It may be hypothesized that weak negative correlations between seminal plasma AOPP and total protein concentrations are associated with the fact that AOPP is derived mainly from oxidation-modified albumin aggregates or fragments [34]. As albumin is the most abundant seminal plasma protein and a part of total protein amount, AOPP formation is reversely associated with the decrease in albumin concentration and, in consequence, with the decrease in total protein levels.

It is worth underlining that, as far as we know, this is the first study in which ROC curves analysis was performed for seminal plasma AOPP, TP and Fe concentrations in the context of decreased male fertility. ROC curves analysis for AOPP revealed this parameter to have moderate clinical value in the differentiation of normozoospermic infertile patients from the asthenoteratozoospermic (proposed cut-off point: 375.75 µmol/L; AUC = 0.837) with a sensitivity of 100% and specificity of 69.7%, and the oligoasthenoteratozoospermic (proposed cut-off point: 254.47 µmol/L; AUC = 0.855) with a sensitivity of 96.4% and specificity of 75.8% (Table 3, Figure 3A,B). Moreover, AOPP concentrations have moderate clinical value in the differentiation of teratozoospermic patients from the asthenoteratozoospermic (proposed cut-off point: 375.75 µmol/L, AUC = 0.838) with a sensitivity of 100% and specificity of 76.7%, and the oligoasthenoteratozoospermic (proposed cut-off point: 268.30 µmol/L, AUC = 0.861) with a sensitivity of 100% and specificity of 80% (Table 3, Figure 3C,D), which led us to conclude that levels of seminal plasma AOPP may be a usable marker of sperm motility disorders (reflected as asthenozoospermia in routine semen analysis). In this study, seminal plasma total protein concentration had a moderate clinical value and enabled differentiation of the AT group from the T group (proposed cut-off point 3.63 g/dL, AUC = 0.728), with a sensitivity of 70% and specificity of 80% (Table 3, Figure 3E), which may suggest that TP levels, similarly to AOPP, also differentiated asthenozoospermia from the other study groups. Seminal plasma iron concentration had moderate clinical value and enabled the differentiation of normozoospermic infertile patients from the oligoasthenoteratozoospermic (proposed cut-off point: 23.88 µg/dL, AUC = 0.725) with a sensitivity of 95.7% and specificity of 50% (Table 3, Figure 3F). Hence, we may presume that the decrease in sperm count is associated with a decreased level of seminal plasma iron.

## 5. Conclusions

**Figure 4:**
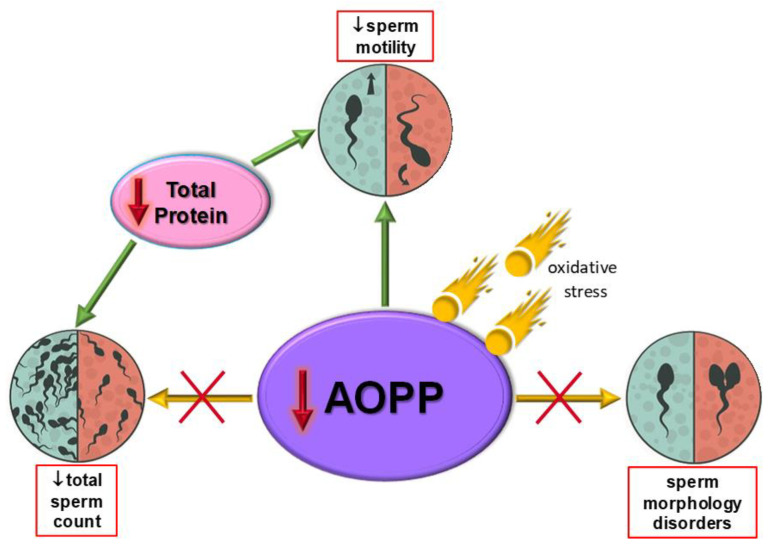
Relationships between the concentrations of advanced oxidation protein products (AOPP) and the levels of markers of oxidative-antioxidant balance in relation to semen parameters.

Oxidative-antioxidant homeostasis is crucial for many physiological processes, including male fertility. Seminal plasma AOPP seems to be a promising parameter differentiating patients with sperm motility problems from men with other sperm disorders; however, the possible association between the formation of advanced oxidation protein products and sperm parameter disorders is multidirectional and may involve additional factors. Our study revealed a significant increase in seminal plasma total protein concentration in the OAT and AT groups in comparison to the T group, suggesting that asthenozoospermia may be connected with seminal plasma total protein levels. The results of our study led us to the conclusion that insufficient iron levels may reflect a decrease in sperm count. The lack of a representative control group of normozoospermic fertile men is the limitation of our study, and makes it impossible to determine if these parameters may be useful in the diagnostics of idiopathic male infertility. Strong positive correlations between seminal plasma concentrations of iron, magnesium, and calcium may confirm that these elements play a very important role in the proper process of spermatozoa maturation, as well as capacitation and acrosomal reaction. The determination of factors associated with oxidative-antioxidant balance may provide valuable information towards the prediction of the effectiveness of AI. As we mentioned in our previous study [21], the antioxidant supplementation of male patients, in appropriate individual doses, may also be worthy of future investigation in the context of AI and IVF. Figure 4 summarizes the main findings from the present research.

## Figures and Tables

**Table 1:** Concentrations of seminal plasma parameters in male patients with fertility disorders.

	Group	N *n* = 33	T *n* = 30	AT *n* = 18	OAT *n* = 28
Parameter		MEAN ± SD Median (Range)	MEAN ± SD Median (Range)	MEAN ± SD Median (Range)	MEAN ± SD Median (Range)
AOPP (µmol/L)		451.89 ± 222.02 483.19 (255.53–552.34) *p* = 0.000035 ^a^ *p* = 0.000002 ^b^	452.70 ± 183.11 505.00 (439.57–554.47) *p* = 0.000043 ^a^ *p* = 0.000002 ^b^	173.79 ± 76.06 163.51 (128.94–206.60)	154.05 ± 61.52 134.26 (110.85–207.13)
TP (g/dL)		3.62 ± 1.12 3.64 (2.69–4.25)	3.18 ± 0.86 3.02 (2.80–3.32) *p* = 0.007072 ^a^ *p* = 0.030200 ^b^	3.94 ± 1.13 4.13 (3.19–4.86)	3.75 ± 0.93 3.66 (2.92–4.26)
Fe (µg/dL)		21.82 ± 10.40 22.01 (14.24–27.57) *p* = 0.031121 ^b^	17.75 ± 6.14 16.44 (14.16–20.47) *p* = 0.020539 ^b^	16.80 ± 3.88 16.15 (13.53–20.14)	14.43 ± 6.18 12.80 (9.82–18.69)
UA (mg/dL)		6.44 ± 2.36 6.58 (4.48–7.34)	5.85 ± 1.26 5.84 (4.91–6.44)	6.28 ± 1.75 5.84 (4.83–7.14)	6.48 ± 1.97 6.47 (4.98–7.42)
Mg (mg/dL)		7.40 ± 2.58 8.25 (5.82–9.41)	6.88 ± 1.97 7.28 (5.01–8.59)	6.68 ± 1.73 6.87 (5.00–7.85)	6.35 ± 2.27 5.80 (4.65–8.54)
Ca (mg/dL)		25.16 ± 13.27 26.79 (12.56–32.86)	21.73 ± 9.66 23.58 (13.03–27.55)	19.38 ± 7.61 18.37 (13.76–23.21)	17.03 ± 10.12 13.95 (9.14–27.81)

AOPP—advanced oxidation protein products, TP—total protein, Fe—iron, UA—uric acid, Mg—magnesium, Ca—calcium, N—normozoospermia, T—teratozoospermia, AT—asthenoteratozoospermia, OAT—oligoasthenoteratozoospermia. Significant differences versus: ^a^ AT group, ^b^ OAT group. A two-tailed *p*-Value < 0.05 was considered significant.

**Table 2:** Significant correlations between concentrations of analyzed parameters.

Compared Parameters	*R*	*p*
Ca & Mg	0.8714	<0.001
Ca & Fe	0.8120	<0.001
Mg & Fe	0.7885	<0.001
AOPP & Fe	0.5393	<0.001
AOPP & Ca	0.5262	<0.001
AOPP & Mg	0.4511	<0.001
AOPP & TP	−0.2650	0.022

Ca—calcium, Mg—magnesium, Fe—iron, AOPP—advanced oxidation protein products, TP—total protein, R—Spearman’s rank coefficient. A two-tailed *p*-value < 0.05 was considered significant.

**Table 3:** Summary of receiver operating characteristic (ROC) curves analysis for seminal plasma parameters.

Parameter	Compared Groups		AUC	AUC with 95% Confidence Interval	Cut Off Point	Sensitivity	Specificity	*p*
AOPP	T	vs. N	0.513	0.368–0.657	482.13	0.667	0.485	0.864
	AT		0.837	0.724–0.949	375.75	1.000	0.697	0.000
	OAT		0.855	0.752–0.958	254.47	0.964	0.758	0.000
	AT vs. T		0.838	0.717–0.959	375.75	1.000	0.767	0.000
	OAT vs. T		0.861	0.755–0.967	268.30	1.000	0.800	0.000
	OAT vs. AT		0.552	0.380–0.723	125.75	0.393	0.778	0.555
TP	T	vs. N	0.364	0.213–0.515	2.76	0.800	0.286	0.077
	AT		0.596	0.429–0.762	4.12	0.550	0.679	0.262
	OAT		0.531	0.373–0.690	2.62	0.958	0.214	0.699
	AT vs. T		0.728	0.571–0.884	3.63	0.700	0.800	0.004
	OAT vs. T		0.674	0.526–0.822	3.47	0.625	0.767	0.021
	OAT vs. AT		0.585	0.410–0.761	4.09	0.708	0.550	0.340
Fe	T	vs. N	0.370	0.150–0.591	12.57	0.889	0.250	0.249
	AT		0.656	0.417–0.894	23.72	1.000	0.500	0.200
	OAT		0.725	0.526–0.923	23.88	0.957	0.500	0.027
	AT vs. T		0.541	0.361–0.721	23.72	1.000	0.185	0.657
	OAT vs. T		0.692	0.539–0.846	14.21	0.696	0.741	0.014
	OAT vs. AT		0.672	0.502–0.843	12.47	0.435	0.933	0.047

AOPP—advanced oxidation protein products, TP—total protein, Fe—iron, N—normozoospermia, T—teratozoospermia, AT—asthenoteratozoospermia, OAT—oligoasthenoteratozoospermia. The area under the ROC curve (AUC) is given with a 95% confidence interval. Data with AUC equal to or higher than 0.725 are marked in grey. Based on the AUC, the clinical value of laboratory tests can be defined as: 0–0.5–zero, 0.5–0.7–limited, 0.7–0.9–moderate, and >0.9–high.

## Data Availability

All data needed to evaluate the conclusions in the article are present in the article. Additional data related to this study are available upon reasonable request from the corresponding author or first author.
